# Maternal psychological distress, education, household income, and congenital heart defects: a prospective cohort study from the Japan environment and children’s study

**DOI:** 10.1186/s12884-021-04001-2

**Published:** 2021-08-07

**Authors:** Yasuaki Saijo, Eiji Yoshioka, Yukihiro Sato, Hiroshi Azuma, Yusuke Tanahashi, Yoshiya Ito, Sumitaka Kobayashi, Machiko Minatoya, Yu Ait Bamai, Keiko Yamazaki, Sachiko Itoh, Chihiro Miyashita, Atsuko Ikeda-Araki, Reiko Kishi, the Japan Environment and Children’s Study (JECS) Group

**Affiliations:** 1 Division of Public Health and Epidemiology, Department of Social Medicine, Asahikawa Medical University, 1-1-1, Midorigaoka-higashi 2-jo, Asahikawa, Hokkaido 078-8510 Japan; 2 Department of Pediatrics, Asahikawa Medical University, Asahikawa, Japan; 3 Faculty of Nursing, Japanese Red Cross Hokkaido College of Nursing, Kitami, Japan; 4 Center for Environmental and Health Sciences, Hokkaido University, Sapporo, Japan

**Keywords:** Congenital heart defects, Psychological distress, Education, Birth cohort

## Abstract

**Background:**

The influence of maternal psychological distress on infant congenital heart defects (CHDs) has not been thoroughly investigated. Furthermore, there have been no reports on the combined effect of maternal psychological distress and socioeconomic status on infant CHDs. This study aimed to examine whether maternal psychological distress, socioeconomic status, and their combinations were associated with CHD.

**Methods:**

We conducted a prospective cohort study using data from the Japan Environment and Children’s Study, which recruited pregnant women between 2011 and 2014. Maternal psychological distress was evaluated using the Kessler Psychological Distress Scale in the first trimester, while maternal education and household income were evaluated in the second and third trimesters. The outcome of infant CHD was determined using the medical records at 1 month of age and/or at birth. Crude- and confounder-adjusted logistic regression analyses were performed to evaluate the association between maternal psychological distress and education and household income on infant CHD.

**Results:**

A total of 93,643 pairs of mothers and infants were analyzed, with 1.1% of infants having CHDs. Maternal psychological distress had a significantly higher odds ratio in the crude analysis but not in the adjusted analysis, while maternal education and household income were statistically insignificant. In the analysis of the combination variable of lowest education and psychological distress, the P for trend was statistically significant in the crude and multivariate model excluding anti-depressant medication, but the significance disappeared in the full model (P = 0.050).

**Conclusions:**

The combination of maternal psychological distress and lower education may be a possible indicator of infant CHD.

**Supplementary Information:**

The online version contains supplementary material available at 10.1186/s12884-021-04001-2.

## Background

Congenital heart defects (CHDs) are the most common non-chromosomal congenital disorders [1] and develop in 0.8 to 1.2% of newborns [2–4]. Although CHD-related mortality has dramatically decreased due to the significant improvements in diagnostics, percutaneous interventions, surgery, and medication, enabling most children to reach adulthood [3], it remains the leading cause of mortality from congenital anomalies and results in a heavy global disease burden [4, 5].

A meta-analysis published in 2014 reported that lower maternal socioeconomic status was slightly associated with increased risk of infant CHDs, while lower maternal education and household income had significantly higher relative risks of 1.11 and 1.05 for infant CHDs, respectively [6]. Furthermore, recent case–control studies have also reported a significant risk of maternal lower education for CHD [7, 8]. A lower socioeconomic status is associated with common mental disorders [9, 10], and the association between anti-depressant use during pregnancy and CHD has been reported in a meta-analysis [11]. Concerning actual depressive symptoms, a case–control study in China reported that depressive symptoms during pregnancy were related to a significantly increased odds ratio (OR) for infant CHDs [12]. However, the diagnosis of depression in a UK cohort and depressive symptoms in a Northern Ireland case–control study was not related to infant CHDs [8, 13].

Thus, whether maternal psychological distress, including depressive symptoms, is a risk factor for CHD has not been fully investigated, and there have been no reports on the combined effects of maternal education, income, and maternal psychological distress on CHD. Further, to our knowledge, there have been no Japanese reports investigating maternal education, income, and maternal psychological distress on CHD risk after adjusting for confounders. This study aimed to examine whether maternal psychological stress, education, household income, and their combinations affected infant CHD using the birth cohort data from the Japan Environment and Children’s Study (JECS) [14, 15].

## Methods

### Participants

The JECS is a nationwide Japanese prospective birth cohort study aiming to identify the environmental factors affecting children’s health and development and is considered to be relatively free of selection bias and representative of the pregnant population in Japan [14, 15]. To cover all the geographical areas of Japan, pregnant women were recruited from 15 Regional Centers (Hokkaido, Miyagi, Fukushima, Chiba, Kanagawa, Koshin, Toyama, Aichi, Kyoto, Osaka, Hyogo, Tottori, Kochi, Fukuoka, and South Kyushu/Okinawa). Baseline recruitment was performed in collaboration with local governments and healthcare providers to maximize representativeness. The children were then followed up until 13 years from birth.

**Fig. 1:**
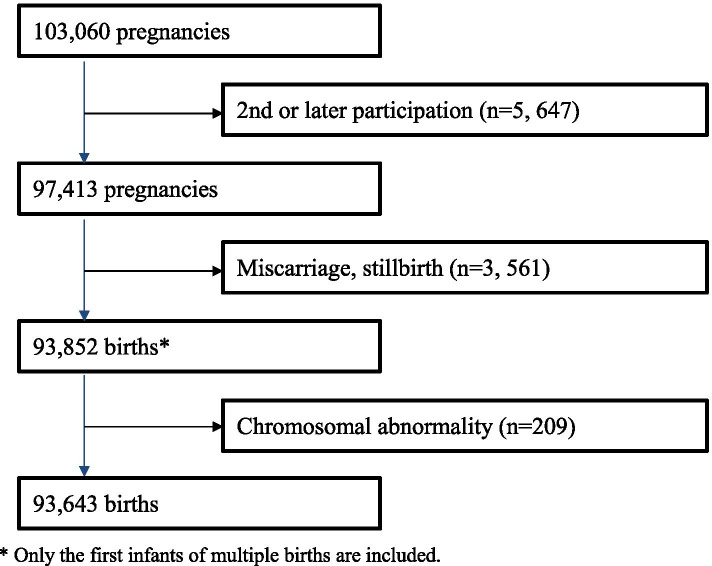
Flowchart of the study. * Only the first infants of multiple births are included

Between January 2011 and March 2014, 103,060 pregnant women in the early stages of pregnancy were recruited. Excluding the pregnancies in the same woman, the study involved 97,413 singleton pregnancies. In our study, we excluded 3,561 pregnancies with a birth status of miscarriage or stillbirth. Of the remaining 93,852 births, which included only the first infant among those with multiple births, those diagnosed with chromosomal abnormalities (n = 209), based on their medical records at birth and 1 month, were excluded. Thus, the final number of participants was 93,643 infants (Fig. 1). Specifically, we used the jecs-ta-20190930 dataset from the JECS, registered in the University Hospital Medical Information Network (UMIN) 000,030,786 (UMIN Clinical Trials Registry, 15/01/2018).

### Ethics statement

The JECS protocol was approved by the Institutional Review Board on Epidemiological Studies of the Ministry of the Environment and by the ethics committees of all participating institutions. This was also conducted in accordance with the Declaration of Helsinki and other nationally valid regulations. Written informed consent was obtained from all participants.

### Outcomes

Infant CHD diagnosed in the medical records at 1 month of age and/or at birth was defined as the outcome. In the sensitivity analyses, the medical records were checked if the caregivers answered positively to the diagnosis of CHD after birth among the infants’ siblings in the 2-year questionnaire. If CHDs were confirmed in the medical records, we defined them as 2-year CHD-positive in the sensitivity analysis.

### Maternal and education, household income, and psychological distress

Socioeconomic status in this study was determined by the mothers’ educational attainment and household income. During pregnancy, questionnaires were distributed to the enrolled mothers during the first (T1; if the participation was delayed, it was distributed during the second/third trimester) and second/third trimesters (T2). The latter included questions about the mothers’ educational attainment, categorized as ≤ 9 years (EDC1: junior high school), 10 to ≤ 12 years (EDC2: high school), 13–15 years (EDC3: technical junior college, technical/vocational college, or associate degree), or ≥ 16 years (EDC4: bachelor’s degree or postgraduate degree). The T2 questionnaire also included questions on household income, categorized as ≤ 199, 200–399, 400–599, 600–799, 800–999, and ≥ 1000 thousand yen. Meanwhile, maternal psychological distress was assessed using the Japanese version of the Kessler 6-Item Psychological Distress Scale (K6) in the T1 questionnaire [16, 17], with a K6 score of > 13 points indicating positive maternal psychological distress [18, 19].

### The other independent variables

Based on previous studies [7–10, 12, 13], the following were selected as covariates: maternal age at delivery, pregnancy body mass index (BMI), paternal education, marital status, mother’s alcohol habit, mother and father’s smoking habits, parity, infant sex, plurality, fertility treatment, hypertensive disorder during pregnancy, thyroid diseases during pregnancy, diabetes mellitus/gestational diabetes during pregnancy, folic acid supplementation at early pregnancy, anti-depressant use at early pregnancy, and mother’s CHDs.

The T1 questionnaire included questions regarding the mother’s birthday, marital status, smoking habit (along with the father’s), folic acid supplementation, anti-depressant use, and history of CHDs. Marital status was classified as married or unmarried, including divorced or bereaved. Smoking habit was categorized as never smoked/quitting smoking before pregnancy or quitting smoking/continued smoking during pregnancy. Folic acid supplementation and anti-depressant use were defined as positive if these were taken between pregnancy perception and 12 weeks of gestation. Lastly, if the mothers responded positively to the query about a previous CHD diagnosis, they were considered positive for CHDs.

On the other hand, the T2 questionnaire inquired about the mothers’ drinking habits. Nondrinkers included those with no history of alcohol intake and those quitting before pregnancy, while drinkers including those currently drinking or quitting during pregnancy.

The following information was also collected from the medical records: infant birth date, plurality, parity, mode of pregnancy (spontaneous, ovulation induction through medication, or artificial insemination/in vitro fertilization), hypertension (hypertension before or during pregnancy), thyroid disease, diabetes mellitus (diabetes mellitus before or during pregnancy), and height and pre-pregnancy weight, from which the BMI was calculated. The mother’s age at infant birth was calculated using her and the infant’s birth dates.

### Statistical analysis

Fisher’s exact test was used to analyze the associations between infant CHD, maternal education, household income, and maternal psychological distress.

For participants with missing data (1.9%), the information was replaced using multiple imputations (25 imputed datasets) based on the assumption that data were missing at random. The imputation model included all the variables analyzed in Fisher’s exact test and the K6 raw score, dichotomized to K6 ≥ 13 or not. Using the imputed datasets, the crude OR of each variable for infant CHD was calculated.

Next, we conducted multivariable logistic regression analyses to estimate the ORs for infant CHD with 95% confidence intervals (95% CIs). First, psychological distress, maternal education, and household income were introduced separately (crude model). In model 1, the three variables were introduced, along with age, pre-pregnancy BMI, father education, marital status, mother drinking habit, mother smoking, paternal smoking, plurality, infant sex, mode of pregnancy, hypertensive disorder, thyroid diseases, diabetes, and folic acid supplementation. Despite the varying reports on anti-depressants being possible mediators of depression [8, 20–23], model 2 was constructed using all the model 1 variables and anti-depressant use.

Then, we constructed the combination variable of the mothers’ lowest education (EDC1) and psychological distress. Lower household income was not included as a combination variable because it had no significant protective OR. We then analyzed the crude and adjusted OR of the combination variable, and the trend P values were calculated using it as an integer variable. P values for the interaction term between the mothers’ lowest education (EDC1) and psychological distress were also analyzed.

As mentioned above, 2-year CHD data was available, but this was restricted to the participants who had answered the 2-year questionnaire (N = 80,468), which may have been biased towards the participants with higher education, higher household income, and lower psychological distress tended to respond to the 2-year questionnaire (Supplemental Table 1). Therefore, in the first sensitivity analysis, 2-year CHD positivity was added to the original CHDs positive outcome, but in the second sensitivity analysis, it was deleted from the original outcome negative.

Two-sided P-values of < 0.05 were considered statistically significant. All analyses were conducted using Stata statistical software version 16.0 for Windows (StataCorp, College Station, TX, USA).

## Results

CHD had a prevalence of 1.1% (Table 1). Among maternal education, household income, and psychological distress, only psychological distress had a statistically significant relationship with infant CHDs in the pairwise deletion analyses (Supplemental Table 2).

**Table 1:** Characteristics of the study population (N = 93,643)

	Number	Percent
Mother’s age at delivery		
-24	9,378	10.0
25–29	25,755	27.5
30–34	33,022	35.3
35–39	21,159	22.6
40-	4,318	4.6
Missing	11	0.0
Pre-pregnancy BMI		
-18.4	14,652	15.7
18.5–24.9	68,683	73.4
25-	10,180	10.9
Missing	128	0.1
Mother’s education		
EDC1	4,389	4.7
EDC2	28,664	30.6
EDC3	38,499	41.1
EDC4	19,910	21.3
Missing	2,181	2.3
Father’s education		
EDC1	6,546	7.0
EDC2	33,325	35.6
EDC3	20,512	21.9
EDC4	30,481	32.6
Missing	2,779	3.0
Household income (10 thousand yen/year)		
-199	4,820	5.2
200–399	29,378	31.4
400–599	28,177	30.1
600–799	13,642	14.6
800–999	5,677	6.1
1000-	3,652	3.9
Missing	8,297	8.9
Marital status		
Married	87,804	93.8
Unmarried, divorced or bereavement	4,189	4.5
Missing	1,650	1.8
Mother’s alcohol intake		
Non-drinker	45,632	48.7
Drinker during early pregnancy	45,604	48.7
Missing	2,407	2.6
Mother’s smoking status		
Non-, ex-smoker	74,878	80.0
Smoker during pregnancy	16,840	18.0
Missing	1,925	2.1
Father’s smoking status		
Non-, ex-smoker	45,760	48.9
Smoker during pregnancy	44,594	47.6
Missing	3,289	3.5
Plurality		
Singleton	92,738	99.0
Multiplet	905	1.0
Parity		
0	38,925	41.6
1	34,151	36.5
> 2	18,183	19.4
Missing	2,384	2.6
Infant’s sex		
Boy	45,587	48.7
Girl	48,038	51.3
Missing	18	0.0
Fertility treatment		
Spontaneous	86,724	92.6
Ovulation induction through medication	2,598	2.8
Artificial insemination or in vitro Fertilization	3,816	4.1
Missing	505	0.5
Hypertensive disorder during pregnancy		
No	88,703	94.7
Yes	3,397	3.6
Missing	1,543	1.7
Thyroid diseases during pregnancy		
No	90,362	96.5
Yes	1,244	1.3
Missing	2,037	2.2
Diabetes mellitus during pregnancy/gestational diabetes		
No	89,160	95.2
Yes	2,940	3.1
Missing	1,543	1.7
Folic acid supplementation at early pregnancy		
No	66,290	70.8
Yes	25,585	27.3
Missing	1,768	1.9
Anti-depressant use		
No	91,651	97.9
Yes	224	0.2
Missing	1,768	1.9
Congenital heart diseases in the mother		
No	92,119	98.4
Yes	289	0.3
Missing	1,235	1.3
Psychological distress in the mother		
No	88,072	94.1
Yes	3,231	3.5
Missing	2,340	2.5
CHD (outcome) in the siblings		
Negative	92,641	98.9
Positive	1,002	1.1

EDC1: junior high school, EDC2: high school, EDC3: technical junior college, technical/vocational college, or EDC4: associate degree bachelor’s degree or postgraduate degree

After multiple imputations in the crude logistic regressions, psychological distress had a significantly higher OR (OR 1.39; 95% CI, 1.03–1.87), while maternal education and household income were statistically insignificant. However, in models 1 and 2, the significance of psychological distress became dismissible (Model 1: OR 1.32; 95% CI, 0.98–1.79; Model2: OR 1.31; 95% CI, 0.97–1.77) (Table 2).

**Table 2:** Crude and adjusted ORs of maternal education, psychological distress, and household income (Multiple imputation, N = 93,643)

		Crude					Model 1					Model 2				
	Proportion^a^	OR	95%CI			P	OR	95%CI			P	OR	95%CI			P
Mother’s education																
EDC1	4.9%	1.24	0.92	-	1.67	0.160	1.09	0.76	-	1.54	0.645	1.08	0.76	-	1.54	0.656
EDC2	31.5%	0.99	0.83	-	1.19	0.927	0.90	0.73	-	1.11	0.334	0.90	0.73	-	1.11	0.332
EDC3	42.0%	1.00	0.84	-	1.18	0.998	0.96	0.80	-	1.15	0.667	0.96	0.80	-	1.15	0.668
EDC4	21.7%	1.00					1.00					1.00				
Household income (10 thousand yen/year)																
-199	5.9%	0.98	0.66	-	1.45	0.915	0.93	0.61	-	1.41	0.721	0.93	0.61	-	1.41	0.718
200–399	35.0%	0.91	0.67	-	1.24	0.542	0.93	0.67	-	1.28	0.652	0.93	0.67	-	1.28	0.650
400–599	32.7%	0.86	0.63	-	1.17	0.334	0.89	0.65	-	1.22	0.466	0.89	0.65	-	1.22	0.467
600–799	15.7%	0.85	0.61	-	1.20	0.360	0.88	0.63	-	1.24	0.462	0.88	0.63	-	1.24	0.464
800–999	6.5%	0.72	0.48	-	1.08	0.116	0.74	0.50	-	1.11	0.148	0.74	0.50	-	1.11	0.148
1000-	4.2%	1.00					1.00					1.00				
Mother’s psychological distress																
No	96.4%	1.00					1.00					1.00				
Yes	3.6%	1.39	1.03	-	1.87	0.029	1.32	0.98	-	1.79	0.066	1.31	0.97	-	1.77	0.080

EDC1: junior high school, EDC2: high school, EDC3: technical junior college, technical/vocational college, or EDC4: associate degree bachelor’s degree or postgraduate degree

^a^Mean proportion of each category in the imputed 25 datasets

Model 1: All listed variables, maternal age, mother BMI, father education, marital status, mother drinking habit, mother smoking, paternal smoking, parity, infant sex, plurality, fertility treatment, hypertensive disorder during pregnancy, thyroid diseases during pregnancy, diabetes mellitus/gestational diabetes during pregnancy, folic acid supplementation during early pregnancy, and mother congenital heart diseases are introduced

Model 2: All variables in Model 1 and anti-depressant use are introduced

In the analysis of the combination variable of maternal psychological distress and lowest education (EDC1), the first and second combinations had no statistical significance, but the trend for p was statistically significant (crude: p = 0.011, Model 1; P = 0.043). However, its significance diminished in Model 2 (p = 0.050) (Table 3, ORs of the combination of EDC1 and psychological distress are shown in Supplemental Table 3). P values for the interaction term between EDC1 and psychological distress had no statistical significance.

**Table 3:** Adjusted ORs of number of maternal education and psychological distress for CHDs (Multiple imputation, 93,643)

		Crude						Model 1						Model 2					
	Proportion^a^	OR	95%CI			P	P for trend	OR	95%CI			P	P for trend	OR	95%CI			P	P for trend
Number of positive^b^																			
0	92.0%	1.00					0.011	1.00					0.043	1.00					0.050
1	7.6%	1.25	1.00	-	1.55	0.052		1.20	0.95	-	1.52	0.123		1.20	0.95	-	1.51	0.135	
2	0.4%	2.02	0.97	-	4.24	0.062		1.87	0.88	-	3.99	0.103		1.83	0.86	-	3.91	0.117	
P for interaction term						0.563						0.573						0.589	

^a^Mean proportion of each category in the imputed 25 datasets

^b^Number of mother junior high school or psychological distress positive

Model 1: All listed variables, maternal age, mother BMI, household income father education, marital status, mother drinking habit, mother smoking, paternal smoking, parity, infant sex, plurality, fertility treatment, hypertensive disorder during pregnancy, thyroid diseases during pregnancy, diabetes mellitus/gestational diabetes during pregnancy, folic acid supplementation during early pregnancy, and mother congenital heart diseases are introduced

Model 2: All variables in Model 1 and anti-depressant use are introduced

P for trend: The number of positives (0–2) was introduced into the model

P for interaction term: Mothers’ lowest education (EDC1), psychological distress, and their interaction term were introduced into the model

In the first sensitivity analysis where the 2-year CHDs positive were added to the original outcome positive, psychological distress had a significantly higher OR only in the crude analysis (OR 1.30; 95% CI, 1.00–1.69) (Supplemental Table 4). A similar result was observed in the second sensitivity analysis where the 2-year CHDs positive were deleted from the original outcome negative (OR 1.39; 95% CI, 1.03–1.87) (Supplemental Table 5). Regarding the combination variable of psychological distress and lowest education, the significant trend for p disappeared in the first sensitivity analysis (Supplemental Table 6) but was still significant in crude and model 1 in the second sensitivity analysis (Supplemental Table 7).

## Discussions

In this prospective birth cohort study, the crude analysis showed that only maternal psychological distress had a significantly higher OR for infant CHDs in the crude analysis, while lower education in mothers and lower household income were unexpectedly insignificant. Further, the combination variable of maternal psychological distress and lowest education had significant trend p values in the crude analysis and Model 1 but not in Model 2.

As previously mentioned, the meta-analysis published in 2014 reported that lower maternal education and household income had significantly higher relative risks for infant CHDs at 1.11 and 1.05, respectively [6]. While the relative risk of household income was significant, its effect size was negligible relative to maternal education. The protective results of higher household income in the present study may be due to the minimal effect size. Furthermore, household income did not necessarily reflect the lifetime socioeconomic status for relatively young women. Education was related to favorable dietary intake patterns among pregnant Japanese women, but the household income was not [24].

In our current study, maternal education had no significant result with the point estimation of crude OR 1.24. Although its OR was higher than household income in the meta-analysis [6], the significance of the relatively low effect size may not be detected due to the lack of statistical power. Moreover, socioeconomic effects on CHDs in developed countries were smaller compared to developing countries [6], and it has been reported that socioeconomic differences affected mortality, morbidity, and risk factors in Japan to a lesser degree than in the US or Europe [25].

A Chinese case–control study found that prenatal depressive symptoms had significantly increased OR (1.94) for CHD. However, the symptoms were evaluated using the Zung Self-Rating Depression Scale after birth, leading to recall bias [12]. Meanwhile, in the UK cohort, diagnosis of depression without anti-depressant use during the first trimester had no significant OR (1.10). Similarly, a case–control study conducted in Northern Ireland reported that having symptoms of “feeling down, depressed or hopeless” within the past month at 10–12 weeks of gestation had no significant OR (1.20) [8, 13]. In the present study, psychological distress had a significant OR on crude analysis, but this became negligible in the adjusted models. The K6 scores in our study could not be directly compared with the previous studies’ because this test evaluates psychological distress and does not focus on depression only [17–19], and the evaluation of depression varied across the studies. However, psychological distress, if present, seemed to have a minor effect on infant CHD.

The combination variable of maternal psychological distress and lowest education (EDC1) had significant trend P values in the crude analysis and Model 1, but both had no statistically significant ORs (2.02, 95% confidence interval: 0.97—4.24) in the crude analysis. Because the effect size of the crude model was relatively large, the combination may identify high-risk pregnancies for infant CHD. The significance of trend p disappeared in Model 2, and the mediation effect of anti-depressants may influence the significance of dilution, although it seemed to be weak [8].

There were doctor-diagnosed child CHDs beyond 2 years among the participants who answered the 2-year age questionnaire, who tended to have a higher socioeconomic status and a lower prevalence of psychological distress [26]. Therefore, to negate this possible bias, we performed two sensitivity analyses. The first analysis included the 2-year CHDs positive and the original CHDs positive outcome, which was almost slightly diluted, as expected. On the other hand, in the second sensitivity analysis, several p values decreased slightly when the 2-year CHDs positive were deleted from the outcome negative. In the main analysis, the relationships were mostly biased to null because the diagnosis of CHDs seemed to result in the nondifferential misclassification of the binary outcomes [27]. Therefore, we believe that the crude effects of psychological distress and the combination variable are small but significant.

Although lower maternal education had no significant effect on infant CHD, the combined effect of maternal psychological distress and lowest education had a significantly increased OR. Therefore, stress and depression screening should be provided for early-stage pregnant women. Education on preventive measures including the use of folate supplementation, minimization of infectious-disease exposure, avoidance of organic solvents, and avoidance of alcohol, tobacco, and illicit drugs [28] may be useful for pregnant women with lower education and psychological distress.

There were several limitations to our present study. First, we did not have adequate statistical power to investigate the weak relationships. For instance, assuming that the non-exposure groups had an average CHD incidence (1.07%), the statistical power to obtain ORs of 1.3 for ECD1 (reference: ECD4) and maternal psychological distress would be 46% and 43%, respectively. Second, our results may not be generalized to other countries due to differences in the educational systems, rates of entering higher educational institutions, and the prevalence of maternal psychological distress. Third, prenatal diagnosis of CHD may affect maternal psychological distress [29]. However, its effect during the first trimester might be limited because the fetal CHDs were mainly diagnosed after 18 to 20 gestational weeks [30], and 80% of the T1 questionnaires were submitted within 20 gestational weeks. Fourth, the severity of CHD was not considered in the analyses. Fifth, although we measured maternal psychological distress using the questionnaire, we did not evaluate specific depressive symptoms.

## Conclusions

The crude analysis in this prospective birth cohort study showed that maternal psychological distress in the first trimester was related to infant CHDs. Meanwhile, the multivariate analysis showed the maternal psychological distress and lowest education were also related. The combination of maternal psychological distress and lowest education may be a possible indicator of infant CHD.

## Supplementary Information

**Additional file 1: Supplemental Table 1**. Education, K6 score, household income of the 2-year questionnaire respondents (N=80,468). *Chi-square test.

**Additional file 2: Supplemental Table 2**. Relationships between the variables and infant congenital heart defect.

**Additional file 3: Supplemental Table 3**. Crude and adjusted ORs of combined maternal education and psychological distress.

**Additional file 4: Supplemental Table 4**. Crude and adjusted ORs of maternal education, psychological distress, and household income with 2-year outcomes.

**Additional file 5: Supplemental Table 5**. Crude and adjusted ORs of maternal education, psychological distress, and household income without 2-y outcomes.

**Additional file 6: Supplemental Table 6**. Adjusted ORs of maternal education and psychological distress (2-y questionnaire outcome added).

**Additional file 7: Supplemental Table 7**. Adjusted ORs of maternal education and psychological distress (only 2-y questionnaire outcome positive excluded).

## Data Availability

Data are unsuitable for public deposition because of the ethical restrictions and legal framework of Japan. Specifically, the Act on the Protection of Personal Information (Act No. 57 of May 30, 2003, amended on September 9, 2015) prohibits the public deposition of data containing personal information. The Ethical Guidelines for Medical and Health Research Involving Human Subjects, enforced by the Japan Ministry of Education, Culture, Sports, Science and Technology and the Ministry of Health, Labour and Welfare, also restrict the open sharing of epidemiologic data. All inquiries about access to data were sent to: jecs-en@nies.go.jp, handled by Dr. Shoji F. Nakayama of the JECS Programme Office, National Institute for Environmental Studies.

## Abbreviations

CHD: Congenital heart defects
JECS: Japan Environment and Children’s Study
EDC: Education
K6: 6-Item Psychological Distress Scale
OR: Odds ratio
CI: Confidence interval

## References

[1] Dolk H, Loane M, Garne E (2010). The prevalence of congenital anomalies in Europe. Adv Exp Med Biol.

[2] Liu Y, Chen S, Zühlke L, Black GC, Choy MK, Li N (2019). Global birth prevalence of congenital heart defects 1970–2017: updated systematic review and meta-analysis of 260 studies. Int J Epidemiol.

[3] Bouma BJ, Mulder BJ (2017). Changing Landscape of Congenital Heart Disease. Circ Res.

[4] Wu W, He J, Shao X. Incidence and mortality trend of congenital heart disease at the global, regional, and national level, 1990–2017. Medicine (Baltimore) 2020;99(23):e20593. 10.1097/MD.0000000000020593 PMC7306355 32502030

[5] Collaborators. GCoD. Global, regional, and national age-sex-specific mortality for 282 causes of death in 195 countries and territories, 1980–2017: a systematic analysis for the Global Burden of Disease Study 2017. Lancet. 2018;392(10159):1736–88. 10.1016/S0140-6736(18)32203-7 PMC6227606 30496103

[6] Yu D, Feng Y, Yang L, Da M, Fan C, Wang S, et al. Maternal socioeconomic status and the risk of congenital heart defects in offspring: a meta-analysis of 33 studies. PLoS One. 2014;9(10):e111056. 10.1371/journal.pone.0111056 PMC4210244 25347676

[7] Arjmandnia M, Besharati M, Rezvan S (2018). Studying the determinant factors leading to congenital heart disease in newborns. J Educ Health Promot.

[8] Dolk H, McCullough N, Callaghan S, Casey F, Craig B, Given J, et al. Risk factors for congenital heart disease: The Baby Hearts Study, a population-based case-control study. PLoS One. 2020;15(2):e0227908. 10.1371/journal.pone.0227908 PMC7039413 32092068

[9] Fryers T, Melzer D, Jenkins R (2003). Social inequalities and the common mental disorders: a systematic review of the evidence. Soc Psychiatry Psychiatr Epidemiol.

[10] Jokela M, Batty GD, Vahtera J, Elovainio M, Kivimäki M (2013). Socioeconomic inequalities in common mental disorders and psychotherapy treatment in the UK between 1991 and 2009. Br J Psychiatry.

[11] Grigoriadis S, VonderPorten EH, Mamisashvili L, Roerecke M, Rehm J, Dennis CL (2013). Antidepressant exposure during pregnancy and congenital malformations: is there an association? A systematic review and meta-analysis of the best evidence. J Clin Psychiatry.

[12] Guo L, Zhao D, Zhang R, Li S, Liu R, Wang H (2019). A Matched Case-Control Study on the Association Between Colds, Depressive Symptoms during Pregnancy and Congenital Heart Disease in Northwestern China. Sci Rep.

[13] Ban L, Gibson JE, West J, Fiaschi L, Sokal R, Smeeth L (2014). Maternal depression, antidepressant prescriptions, and congenital anomaly risk in offspring: a population-based cohort study. BJOG.

[14] Kawamoto T, Nitta H, Murata K, Toda E, Tsukamoto N, Hasegawa M, et al. Rationale and study design of the Japan environment and children’s study (JECS). BMC Public Health. 2014;14:25. 10.1186/1471-2458-14-25 PMC3893509 24410977

[15] Michikawa T, Nitta H, Nakayama SF, Yamazaki S, Isobe T, Tamura K, et al. Baseline Profile of Participants in the Japan Environment and Children’s Study (JECS). J Epidemiol. 2018;28(2):99–104. 10.2188/jea.JE20170018 PMC5792233 29093304

[16] Kessler RC, Andrews G, Colpe LJ, Hiripi E, Mroczek DK, Normand SL (2002). Short screening scales to monitor population prevalences and trends in non-specific psychological distress. Psychol Med.

[17] Furukawa TA, Kawakami N, Saitoh M, Ono Y, Nakane Y, Nakamura Y (2008). The performance of the Japanese version of the K6 and K10 in the World Mental Health Survey Japan. Int J Methods Psychiatr Res.

[18] Prochaska JJ, Sung HY, Max W, Shi Y, Ong M (2012). Validity study of the K6 scale as a measure of moderate mental distress based on mental health treatment need and utilization. Int J Methods Psychiatr Res.

[19] Kessler RC, Barker PR, Colpe LJ, Epstein JF, Gfroerer JC, Hiripi E (2003). Screening for serious mental illness in the general population. Arch Gen Psychiatry.

[20] Wurst KE, Poole C, Ephross SA, Olshan AF (2010). First trimester paroxetine use and the prevalence of congenital, specifically cardiac, defects: a meta-analysis of epidemiological studies. Birth Defects Res A Clin Mol Teratol.

[21] Myles N, Newall H, Ward H, Large M (2013). Systematic meta-analysis of individual selective serotonin reuptake inhibitor medications and congenital malformations. Aust N Z J Psychiatry.

[22] Huybrechts KF, Palmsten K, Avorn J, Cohen LS, Holmes LB, Franklin JM (2014). Antidepressant use in pregnancy and the risk of cardiac defects. N Engl J Med.

[23] Anderson KN, Lind JN, Simeone RM, Bobo WV, Mitchell AA, Riehle-Colarusso T (2020). Maternal Use of Specific Antidepressant Medications During Early Pregnancy and the Risk of Selected Birth Defects. JAMA Psychiat.

[24] Murakami K, Miyake Y, Sasaki S, Tanaka K, Ohya Y, Hirota Y (2009). Education, but not occupation or household income, is positively related to favorable dietary intake patterns in pregnant Japanese women: the Osaka Maternal and Child Health Study. Nutr Res.

[25] Kagamimori S, Gaina A, Nasermoaddeli A (2009). Socioeconomic status and health in the Japanese population. Soc Sci Med.

[26] Kigawa M, Tsuchida A, Matsumura K, Takamori A, Ito M, Tanaka T, et al. Factors of non-responsive or lost-to-follow-up Japanese mothers during the first year post partum following the Japan Environment and Children's Study: a longitudinal cohort study. BMJ Open. 2019;9(11):e031222. 10.1136/bmjopen-2019-031222 PMC6858228 31722943

[27] Rothman KJ, Greenland S, Lash TL. Modern Epidemiology. Philadelphia: Lippincott Williams & Wilkins; 2012.

[28] Patel SS, Burns TL (2013). Nongenetic risk factors and congenital heart defects. Pediatr Cardiol.

[29] Rychik J, Donaghue DD, Levy S, Fajardo C, Combs J, Zhang X (2013). Maternal psychological stress after prenatal diagnosis of congenital heart disease. J Pediatr.

[30] Satomi G (2015). Guidelines for fetal echocardiography. Pediatr Int.

