# Supplementary material for: Maternal psychological distress, education, household income, and congenital heart defects: a prospective cohort study from the Japan environment and children’s study
Source: BMC Pregnancy Childbirth. 2021 Aug 7;21:544. doi: 10.1186/s12884-021-04001-2 (PMC8348993; doi:10.1186/s12884-021-04001-2)
Supplement: Supplementary file 1 — Additional file 1: Supplemental Table 1. Education, K6 score, household income of the 2-year questionnaire respondents (N=80,468). *Chi-square test. [file 12884_2021_4001_MOESM1_ESM.docx]

Supplemental Table 1 Education, K6 score, household income of the 2-year questionnaire respondents (N=80,468)

*Chi-square test

|  | 2-year questionnaire respondents | |  | Analyzed participants | |
| --- | --- | --- | --- | --- | --- |
|  | (N=80,468) | |  | (N=93,643) | |
|  | N | % |  | N | % |
| Mother’s education |  |  |  |  |  |
| EDC1 | 3,096 | 3.9 |  | 4,389 | 4.7 |
| EDC2 | 24,059 | 29.9 |  | 28,664 | 30.6 |
| EDC3 | 34,092 | 42.4 |  | 38,499 | 41.1 |
| EDC4 | 18,267 | 22.7 |  | 19,910 | 21.3 |
| Missing | 954 | 1.2 |  | 2,181 | 2.3 |
| Household income (10 thousand yen/year) |  |  |  |  |  |
| -199 | 3,785 | 4.7 |  | 4,820 | 5.2 |
| 200-399 | 25,287 | 31.4 |  | 29,378 | 31.4 |
| 400-599 | 24,894 | 30.9 |  | 28,177 | 30.1 |
| 600-799 | 12,251 | 15.2 |  | 13,642 | 14.6 |
| 800-999 | 5,102 | 6.3 |  | 5,677 | 6.1 |
| 1000- | 3,261 | 4.1 |  | 3,652 | 3.9 |
| Missing | 5,888 | 7.3 |  | 8,297 | 8.9 |
| Mother’s psychological distress |  |  |  |  |  |
| No | 76,519 | 95.1 |  | 88,072 | 94.1 |
| Yes | 2,624 | 3.3 |  | 3,231 | 3.5 |
| Missing | 1,325 | 1.7 |  | 2,340 | 2.5 |

EDC1: junior high school, EDC2: high school, EDC3: technical junior college, technical/vocational college, or EDC4: associate degree bachelor’s degree or postgraduate degree.
