# Supplementary material for: Maternal psychological distress, education, household income, and congenital heart defects: a prospective cohort study from the Japan environment and children’s study
Source: BMC Pregnancy Childbirth. 2021 Aug 7;21:544. doi: 10.1186/s12884-021-04001-2 (PMC8348993; doi:10.1186/s12884-021-04001-2)
Supplement: Supplementary file 2 — Additional file 2: Supplemental Table 2. Relationships between the variables and infant congenital heart defect. [file 12884_2021_4001_MOESM2_ESM.docx]

Supplemental Table 2 Relationships between the variables and infant congenital heart defect

|  | No CHD | | CHD | | P |
| --- | --- | --- | --- | --- | --- |
|  | N | % | N | % |  |
| Mothers' education |  |  |  |  | 0.409 |
| EDC1 | 4,333 | 98.7 | 56 | 1.28 |  |
| EDC2 | 28,371 | 99.0 | 293 | 1.02 |  |
| EDC3 | 38,097 | 99.0 | 402 | 1.04 |  |
| EDC4 | 19,702 | 99.0 | 208 | 1.04 |  |
| Household income (thousand yen/year) |  |  |  |  | 0.518 |
| -199 | 4,764 | 98.8 | 56 | 1.16 |  |
| 200-399 | 29,057 | 98.9 | 321 | 1.09 |  |
| 400-599 | 27,885 | 99.0 | 292 | 1.04 |  |
| 600-799 | 13,500 | 99.0 | 142 | 1.04 |  |
| 800-999 | 5,628 | 99.1 | 49 | 0.86 |  |
| 1000- | 3,607 | 98.8 | 45 | 1.23 |  |
| Psychological distress in the mother |  |  |  |  | 0.036 |
| No | 87,145 | 99.0 | 927 | 1.05 |  |
| Yes | 3,184 | 98.6 | 47 | 1.45 |  |

EDC1: junior high school, EDC2: high school, EDC3: technical junior college, technical/vocational college, or EDC4: associate degree bachelor’s degree or postgraduate degree.
