# Supplementary material for: Maternal psychological distress, education, household income, and congenital heart defects: a prospective cohort study from the Japan environment and children’s study
Source: BMC Pregnancy Childbirth. 2021 Aug 7;21:544. doi: 10.1186/s12884-021-04001-2 (PMC8348993; doi:10.1186/s12884-021-04001-2)
Supplement: Supplementary file 3 — Additional file 3: Supplemental Table 3. Crude and adjusted ORs of combined maternal education and psychological distress. [file 12884_2021_4001_MOESM3_ESM.docx]

Supplemental Table 3 Crude and adjusted ORs of combined maternal education and psychological distress

|  | Proportion* | OR | 95%CI | | | P |  | OR | 95%CI | | | P |  | OR | 95%CI | | | P |
| --- | --- | --- | --- | --- | --- | --- | --- | --- | --- | --- | --- | --- | --- | --- | --- | --- | --- | --- |
| Psychological distress(-) + ECD2-4 | 92.0% | 1.00 |  |  |  |  |  | 1.00 |  |  |  |  |  | 1.00 |  |  |  |  |
| Psychological distress(-) + ECD1 | 4.5% | 1.19 | 0.88 | - | 1.60 | 0.253 |  | 1.14 | 0.83 | - | 1.57 | 0.420 |  | 1.14 | 0.83 | - | 1.57 | 0.425 |
| Psychological distress(+) + ECD2-4 | 3.2% | 1.32 | 0.96 | - | 1.83 | 0.088 |  | 1.28 | 0.92 | - | 1.77 | 0.138 |  | 1.27 | 0.91 | - | 1.75 | 0.157 |
| Psychological distress(+) + ECD1 | 0.4% | 2.02 | 0.97 | - | 4.24 | 0.062 |  | 1.86 | 0.87 | - | 3.97 | 0.107 |  | 1.82 | 0.85 | - | 3.89 | 0.121 |

Junior high school: EDC1

*Mean proportion of each category in the imputed 25 datasets

(Multiple imputation, N=93,643)

Model 1: All listed variables, maternal age, mother BMI, household income, father education, marital status, mother drinking habit, mother smoking, paternal smoking, parity, infant sex, plurality, fertility treatment, hypertensive disorder during pregnancy, thyroid diseases during pregnancy, diabetes mellitus during pregnancy /gestational diabetes, folic acid supplementation during early pregnancy, and mother congenital heart diseases are introduced.

Model 2: All the variables in Model 1 and anti-depressant intake are introduced.
