# Supplementary material for: Maternal psychological distress, education, household income, and congenital heart defects: a prospective cohort study from the Japan environment and children’s study
Source: BMC Pregnancy Childbirth. 2021 Aug 7;21:544. doi: 10.1186/s12884-021-04001-2 (PMC8348993; doi:10.1186/s12884-021-04001-2)
Supplement: Supplementary file 5 — Additional file 5: Supplemental Table 5. Crude and adjusted ORs of maternal education, psychological distress, and household income without 2-y outcomes. [file 12884_2021_4001_MOESM5_ESM.docx]

Supplemental Table 5 Crude and adjusted ORs of maternal education, psychological distress, and household income without 2-y outcomes

|  |  | Crude | | | | |  | Model 1 | | | | |  | Model 2 | | | | |
| --- | --- | --- | --- | --- | --- | --- | --- | --- | --- | --- | --- | --- | --- | --- | --- | --- | --- | --- |
|  | Proportion* | OR | 95%CI | | | P |  | OR | 95%CI |  |  | P |  | OR | 95%CI | | | P |
| Mother’s education |  |  |  |  |  |  |  |  |  |  |  |  |  |  |  |  |  |  |
| EDC1 | 4.9% | 1.24 | 0.92 | - | 1.67 | 0.164 |  | 1.08 | 0.76 | - | 1.54 | 0.657 |  | 1.08 | 0.76 | - | 1.53 | 0.668 |
| EDC2 | 31.5% | 0.99 | 0.83 | - | 1.19 | 0.935 |  | 0.90 | 0.73 | - | 1.11 | 0.334 |  | 0.90 | 0.73 | - | 1.11 | 0.332 |
| EDC3 | 42.0% | 1.00 | 0.85 | - | 1.18 | 1.000 |  | 0.96 | 0.80 | - | 1.15 | 0.665 |  | 0.96 | 0.80 | - | 1.15 | 0.666 |
| EDC4 | 21.7% | 1.00 |  |  |  |  |  | 1.00 |  |  |  |  |  | 1.00 |  |  |  |  |
| Household income (10 thousand yen/year) | | |  |  |  |  |  |  |  |  |  |  |  |  |  |  |  |  |
| -199 | 5.9% | 0.98 | 0.66 | - | 1.45 | 0.918 |  | 0.93 | 0.61 | - | 1.41 | 0.728 |  | 0.93 | 0.61 | - | 1.41 | 0.725 |
| 200-399 | 35.0% | 0.91 | 0.67 | - | 1.24 | 0.548 |  | 0.93 | 0.67 | - | 1.29 | 0.661 |  | 0.93 | 0.67 | - | 1.29 | 0.659 |
| 400-599 | 32.7% | 0.86 | 0.63 | - | 1.17 | 0.337 |  | 0.89 | 0.65 | - | 1.22 | 0.472 |  | 0.89 | 0.65 | - | 1.22 | 0.473 |
| 600-799 | 15.7% | 0.85 | 0.61 | - | 1.20 | 0.364 |  | 0.88 | 0.63 | - | 1.24 | 0.466 |  | 0.88 | 0.63 | - | 1.24 | 0.468 |
| 800-999 | 6.5% | 0.72 | 0.48 | - | 1.08 | 0.116 |  | 0.74 | 0.50 | - | 1.11 | 0.149 |  | 0.74 | 0.50 | - | 1.11 | 0.150 |
| 1000- | 4.2% | 1.00 |  |  |  |  |  | 1.00 |  |  |  |  |  | 1.00 |  |  |  |  |
| Psychological distress in the mother |  |  |  |  |  |  |  |  |  |  |  |  |  |  |  |  |  |  |
| No | 96.4% | 1.00 |  |  |  |  |  | 1.00 |  |  |  |  |  | 1.00 |  |  |  |  |
| Yes | 3.6% | 1.39 | 1.03 | - | 1.87 | 0.029 |  | 1.32 | 0.98 | - | 1.79 | 0.066 |  | 1.31 | 0.97 | - | 1.77 | 0.080 |

EDC1: junior high school, EDC2: high school, EDC3: technical junior college, technical/vocational college, or EDC4: associate degree bachelor’s degree or postgraduate degree.

(Multiple imputation, N=93,269)

*Mean proportion of each category in the imputed 25 datasets

Model 1: All listed variables, maternal age, mother BMI, father education, marital status, mother drinking habit, mother smoking, paternal smoking, parity, infant sex, plurality, fertility treatment, hypertensive disorder during pregnancy, thyroid diseases during pregnancy, diabetes mellitus/gestational diabetes during pregnancy, folic acid supplementation during early pregnancy, and mother congenital heart diseases are introduced.

Model 2: All the variables in Model 1 and anti-depressant intake are introduced.
