# Supplementary material for: Maternal psychological distress, education, household income, and congenital heart defects: a prospective cohort study from the Japan environment and children’s study
Source: BMC Pregnancy Childbirth. 2021 Aug 7;21:544. doi: 10.1186/s12884-021-04001-2 (PMC8348993; doi:10.1186/s12884-021-04001-2)
Supplement: Supplementary file 7 — Additional file 7: Supplemental Table 7. Adjusted ORs of maternal education and psychological distress (only 2-y questionnaire outcome positive excluded). [file 12884_2021_4001_MOESM7_ESM.docx]

Supplemental Table 7 Adjusted ORs of maternal education and psychological distress (only 2-y questionnaire outcome positive excluded)

|  |  | Crude | | | | | |  | Model 1 | | | | | |  | Model 2 | | | | | |  |
| --- | --- | --- | --- | --- | --- | --- | --- | --- | --- | --- | --- | --- | --- | --- | --- | --- | --- | --- | --- | --- | --- | --- |
|  | Proportion* | OR | 95%CI | | | P | P for trend |  | OR | 95%CI | | | P | P for trend |  | OR | 95%CI | | | P | P for trend |  |
| Number of positive |  |  |  |  |  |  |  |  |  |  |  |  |  |  |  |  |  |  |  |  |  | |
| 0 | 92.0% | 1.00 |  |  |  |  | 0.011 |  | 1.00 |  |  |  |  | 0.044 |  | 1.00 |  |  |  |  | 0.052 | |
| 1 | 7.6% | 1.24 | 1.00 | - | 1.55 | 0.053 |  |  | 1.20 | 0.95 | - | 1.52 | 0.126 |  |  | 1.19 | 0.94 | - | 1.51 | 0.139 |  | |
| 2 | 0.4% | 2.02 | 0.96 | - | 4.22 | 0.063 |  |  | 1.87 | 0.88 | - | 3.97 | 0.106 |  |  | 1.82 | 0.86 | - | 3.89 | 0.120 |  | |
| P for interaction term |  |  |  |  |  | 0.567 |  |  |  |  |  |  | 0.576 |  |  |  |  |  |  | 0.592 |  | |

*Mean proportion of each category in the imputed 25 datasets

(Multiple imputation, N=93,269)

Model 1: All listed variables, maternal age, mother BMI, household income father education, marital status, mother drinking habit, mother smoking, paternal smoking, parity, infant sex, plurality, fertility treatment, hypertensive disorder during pregnancy, thyroid diseases during pregnancy, diabetes mellitus/gestational diabetes during pregnancy, folic acid supplementation during early pregnancy, and mother congenital heart diseases are introduced.

Model 2: All the variables in Model 1 and anti-depressant intake are introduced.

P for trend: The number of positives (0-2) is introduced into the model.

P for interaction term: Mothers’ lowest education (EDC1), psychological distress, and their interaction term were introduced into the model.
